# Seasonal variations in *Plasmodium falciparum* parasite prevalence assessed by varying diagnostic tests in asymptomatic children in southern Ghana

**DOI:** 10.1371/journal.pone.0199172

**Published:** 2018-06-15

**Authors:** Ruth Ayanful-Torgby, Neils B. Quashie, Johnson N. Boampong, Kim C. Williamson, Linda E. Amoah

**Affiliations:** 1 Department of Immunology, Noguchi Memorial Institute for Medical Research, University of Ghana, Accra, Ghana; 2 School of Biomedical Sciences, University of Cape Coast, Cape Coast, Ghana; 3 Centre for Tropical Clinical Pharmacology and Therapeutics, University of Ghana, Accra, Ghana; 4 Department of Microbiology, Uniform Services University of the Health Sciences, Bethesda, Maryland, United States of America; Centro de Pesquisas Rene Rachou, BRAZIL

## Abstract

*Plasmodium falciparum* infections presenting either as symptomatic or asymptomatic may contain sexual stage parasites (gametocytes) that are crucial to malaria transmission. In this study, the prevalence of microscopic and submicroscopic asexual and gametocyte parasite stages were assessed in asymptomatic children from two communities in southern Ghana. Eighty children aged twelve years and below, none of whom exhibited signs of clinical malaria living in Obom and Cape Coast were sampled twice, one during the rainy (July 2015) and subsequently during the dry (January 2016) season. Venous blood was used to prepare thick and thin blood smears, spot a rapid malaria diagnostic test (PfHRP2 RDT) as well as prepare filter paper blood spots. Blood cell pellets were preserved in Trizol for RNA extraction. Polymerase chain reaction (PCR) and semi-quantitative real time reverse transcriptase PCR (qRT-PCR) were used to determine submicroscopic parasite prevalence. In both sites 87% (95% CI: 78–96) of the asymptomatic individuals surveyed were parasites positive during the 6 month study period. The prevalence of asexual and gametocyte stage parasites in the rainy season were both significantly higher in Obom than in Cape Coast (P < 0.001). Submicroscopic gametocyte prevalence was highest in the rainy season in Obom but in the dry season in Cape Coast. Parasite prevalence determined by PCR was similar to that determined by qRT-PCR in Obom but significantly lower than that determined by qRT-PCR in Cape Coast. Communities with varying parasite prevalence exhibit seasonal variations in the prevalence of gametocyte carriers. Submicroscopic asymptomatic parasite and gametocyte carriage is very high in southern Ghana, even during the dry season in communities with low microscopic parasite prevalence and likely to be missed during national surveillance exercises.

## Introduction

For malaria elimination and eradication to be possible, control policies for all the various manifestations of malaria, including asymptomatic infections are needed. Currently, the majority of malaria elimination efforts include the use of insecticide treated nets and spray that is vector targeted. Seasonal chemoprophylaxis is also administered predominantly in high risk populations [1]. However, to move towards malaria elimination, an accurate profile of malaria transmission dynamics and infecting parasite clones in different transmission settings are needed to inform the implementation of the aforementioned strategies [2,3]. Inadequate information on malaria transmission intensity in endemic areas is common [4,5], and has resulted in either under or over estimation of the true prevalence of *Plasmodium* infections within different communities [6]. Unfortunately, the true prevalence of *Plasmodium falciparum* (*P*. *falciparum)* infections in a population is often not available, either because the tools used in the measurements lacked the requisite sensitivity or those specific sites were not included in the study. The results obtained from the sites used in the study are usually generalized for nearby communities [7,8]. The use of molecular methods to monitor parasite prevalence and provide an accurate mapping of transmission intensity will help address the above limitations on inadequate infection prevalence in most malaria endemic area. Although a number of molecular tools have been developed they need to be tested in the field to appreciate their strengths and limitations.

In order to achieve continuous and effective control of malaria to eliminate the burden associated with the disease [9], all the various presentations of *Plasmodium* infections including microscopic or submicroscopic and symptomatic or asymptomatic infections must be targeted. Asymptomatic infections are predominantly submicroscopic but still have the potential to influence malaria transmission [10]. Seasonal malaria chemoprevention (SMC) interventions are usually implemented in high parasite prevalence settings [1,11–13]. This intervention has been successfully deployed in Northern Ghana, where parasite prevalence is high (>40%) and malaria transmission is seasonal [14,15]. However, evidence from other studies show that transmission hotspots exist in areas generally considered as low transmission settings. These hotspots in the low transmission setting have similar high (>40%) parasite prevalence as in high transmission settings but are usually not captured for SMC or other malaria control interventions. These communities are often close to water bodies or have poor sanitation systems [16,17] and harbour transmission reservoirs that sustain the spread of malaria. Recent discussions have suggested an extension of SMC [18] to low transmission settings [19]. Such control measures have the potential to reduce *P*. *falciparum* parasite infections in communities with varying parasite prevalence, but need to be effectively monitored. Variations in infecting parasite densities and prevalence may influence the effectiveness of implemented interventions which requires careful selection of tools to monitor parasite prevalence amongst other factors [20,21].

As a first step towards accurately mapping parasite prevalence to designing a malaria elimination program we evaluated the relationship between asexual parasite and gametocyte prevalence in asymptomatic children living in two communities with varied malaria transmission patterns in Ghana. We utilized tools with varying sensitivities to assess the seasonal variations in *P*. *falciparum* parasites prevalence and have evidence to suggest the most appropriate tool to use to assess parasite prevalence in these two distinct settings.

## Methods

### Ethics, study site, population and sampling

The study had approval from the Institutional Review Board of the Noguchi Memorial Institute for Medical Research (NMIMR) and Ghana Health Services. Participants were enrolled only after written parental consent had been obtained.

The study randomly enrolled children from public primary schools in Obom and Cape Coast, both in southern Ghana. Obom (05°34′ N, 0° 20′ W), is a rural setting in Greater Accra and also lies in the Coastal savannah region. The mean annual rainfall varies between 790 mm to 1270 mm, along the coast to the extreme north. Relative humidity is about 75% between February and March and the main economic activity in Obom is farming. Malaria transmission is perennial with most of the disease occurring during the major rainy season in June/July [22,23]. Cape Coast (05°05' N, 01° 15' W), is an urban setting and lies in the Coastal savannah region on the Gulf of Guinea. The major rainy season is between May and July with mean monthly relative humidity varying between 85 and 99% and the main economic activity of the indigenes is fishing and farming. Malaria transmission in Cape Coast is seasonal with most of the disease occurring during June to July.

Two cross sectional surveys, involving 80 school children (40 from each site) aged between 6 and 12 years were conducted in the peak / rainy season (July, 2015) and during the off peak /dry season (January, 2016) after obtaining written parental consent. On each visit, body temperature was measured using a digital thermometer, after which 2.5 mL of venous blood was collected into EDTA vacutainer tubes and an aliquot used to prepare filter paper (Whatman^®^ 3 mm) blood blots, thick and thin blood smears and spot histidine rich 2 protein malaria rapid diagnostic test (RDT). The rest of the blood sample was immediately separated into plasma and blood cells. One hundred microliters of the blood cells were preserved in 500 μL of Trizol (Invitrogen, UK) and the plasma preserved at -20 °C. During the dry season, haemoglobin levels were assessed using the Urit-12 haemoglobin meter.

### *Plasmodium falciparum* parasite detection

*Plasmodium falciparum* species identification and parasitaemia were determined using 100X oil immersion microscopy. The thin and thick blood smears were processed for Giemsa staining and evaluated using a WHO protocol [24]. *Plasmodium* species were identified after evaluating the thin films and parasite density was estimated using the thick films. Parasite density was determined as the percent of infected erythrocytes counted per 200 white blood cells (WBC) based on a WHO protocol [24].

### Parasite DNA extraction

Genomic DNA was extracted from two 3 mm punched discs of dried filter paper blood blots using the Chelex extraction protocol [25]. Briefly, the two punched discs for each sample were incubated in 1 mL of PBS solution for 10 minutes. The samples were washed twice with 1 mL PBS, followed by centrifugation at 14,000 rpm for 2 minutes. DNA was then extracted from the discs with 100 μL of 10% Chelex (Sigma-Aldrich, USA) in nuclease-free water by heating at 99 °C for 10 minutes, with occasional vortexing. Finally the sample was centrifuges at 14,000 rpm for 1 minute and the supernatant containing the DNA aliquoted into a new tube and stored at 20 °C for PCR.

### PCR identification of *Plasmodium falciparum* parasites

Submicroscopic *P*. *falciparum* parasites were estimated based on the amplification of the *18S rRNA* gene. Nested PCR was performed using genus and species specific primers as described by Singh *et al*. [26]. All reactions were carried out in a 20 μL volume containing 200 nM dNTP, 2 mM MgCl_2_, 200 nM of each primer, and 0.5 U of One Taq DNA polymerase (New England BioLAB, UK). Four microliters (>50 ng) of gDNA was used as the template for the primary reaction and 2 μL of the primary reaction product was used a template for the secondary reaction. The reaction cycling conditions were: initial denaturation at 94 °C for 5 minutes, followed by 30 cycles at 94 °C for 1 minute denaturation; annealing at 50 °C (55 °C for nest 2) for 30 seconds, and 68 °C for 30 seconds; with final extension at 68 °C for 5 minutes. The PCR reaction mixtures were run on a thermal cycler (BioMetra T3000, Germany). Positive and negative controls for the PCR reactions comprising of the 3D7 *P*. *falciparum* strain and a no template control respectively were included in each set of the reactions. Amplified PCR products were visualized under UV illumination after electrophoretic separation on a 2% ethidium bromide stained agarose gel.

### Parasite RNA extraction, purification and RT-PCR analysis

Total RNA was extracted from 80 paired trizol preserved samples collected in the rainy and dry seasons in both sites using the RNeasy micro kit (Qiaqen, USA) following the manufacturer’s protocol. Complementary DNA (cDNA) was prepared using ProtoScript^®^ First Strand cDNA Synthesis Kit (New England BioLab, UK) and a mix of 3 μM of random hexamers and 2.5 μM oligo (dT) primers. gDNA contamination check on the extracted RNA was done as previously reported [27]. The cDNA samples were diluted (1:20) before triplicate run with fast SYBR^®^ Green 2X master mix RT-PCR kit on a QuantStudio 3^™^ Real-Time PCR System (Thermo Scientific, USA). The Fast cycling condition (95 °C for 20 sec, 40 cycles of 95 °C for 1 sec and 60 °C for 20 sec) was used for qRT-PCR amplifications. Real time RT-PCR was carried out on cDNA samples to assess submicroscopic asexual parasite as well as mature gametocyte carriage using *Pf18S rRNA* and *Pfs25* transcript levels respectively. cDNA prepared from ring stage parasites (asexual) as well as matured gametocytes from *P*. *falciparum* NF54 parasite served as controls for both qRT-PCR reactions and primers were validated as previously described [27].

### Data analysis

Statistical analysis was performed using Mann-Whitney paired two tailed t-test (GraphPad Prism v5.0) for the age groups and Two-sample test for equality of proportions with continuity correction (R version 3.4) to determine associations between parasite prevalence within and between participants in the two communities. The qRT-PCR data was analyzed with Quanti v1.3.1 Software (Thermo Scientific, USA). The threshold cycle (CT) cut off based on the negative and the positive controls as previously described [27], where it was used to classified the samples as negative or positive for asexual parasites and gametocytes. Tests were considered statistically significant when P values were < 0.05.

## Results

### Clinical characteristics of the study participants

The clinical characteristics of the participants in Obom and Cape Coast are shown in Table 1. The mean age of participants in both sites was not significantly different (P = 0.48, Mann Whitney paired two tailed test). In Obom the mean parasite density estimated from the thick blood smears increased from 1753 ± 1476 parasites/μL of whole blood in the 22 smear positive samples out of 34 total samples in the rainy season to 2344 ± 2636 parasites/μL of whole blood in the 11 smear positive samples out of 40 total samples during the dry season, although fewer individuals were infected. In Cape Coast, the mean parasite density was 1015 ± 971 parasites/μL of whole blood in the four smear positive samples out of a total of 39 samples in the rainy season, which decreased during the dry season to 530 ± 561 parasites/μL of whole blood in the two smear positive samples out of a total of 36 samples. The HRP2 RDT test was only performed in the dry season with 65% (95% CI: 46%–80%) positivity rate in Obom, significantly higher (P < 0.0001, Two-sample test for equality of proportions) than the 35% (95% CI: 20%–53%) recorded in Cape Coast.

**Table 1:** Clinical characteristics of the study participants.

Parameter	Obom		Cape Coast	
	July 2015	January 2016*	July 2015	January 2016
Temperature °C				
Mean	36.51	36.35	36.46	36.49
SD	0.51	0.59	0.53	0.53
Range	(35.20–37.60)	(35.20–37.20)	(34.40–37.40)	(34.90–37.30)
Haemoglobin (g/dL)				
Mean	^	10.59	^	11.87
SD		2.09		1.43
Range		(4.46^#^–14.00)		(7.50–14.60)
Parasite density/μL of blood				
Mean	1753	2344	1015	530
SD	1476	2636	971	561
Range	(160–5040)	(560–6920)	(80–3038)	(80–530)
Microscopy				
Positive (p/n)	22/34	11/40	4/39	2/36
HRP2 (RDT)				
Positive %	^	65	^	36
95% CI (%)		50–80		20–53

^ Not done;

^#^ A participant had sickle cell disease; p, number that were microscopy positive; n, number of participant tested in the group;

*Malaria control intervention was implemented in the community four (4) months before sampling. There were 32 and 29 children in Obom and Cape Coast respectively who were present at both time points (paired samples).

### *Plasmodium falciparum* asexual parasite prevalence

**Fig 1:**
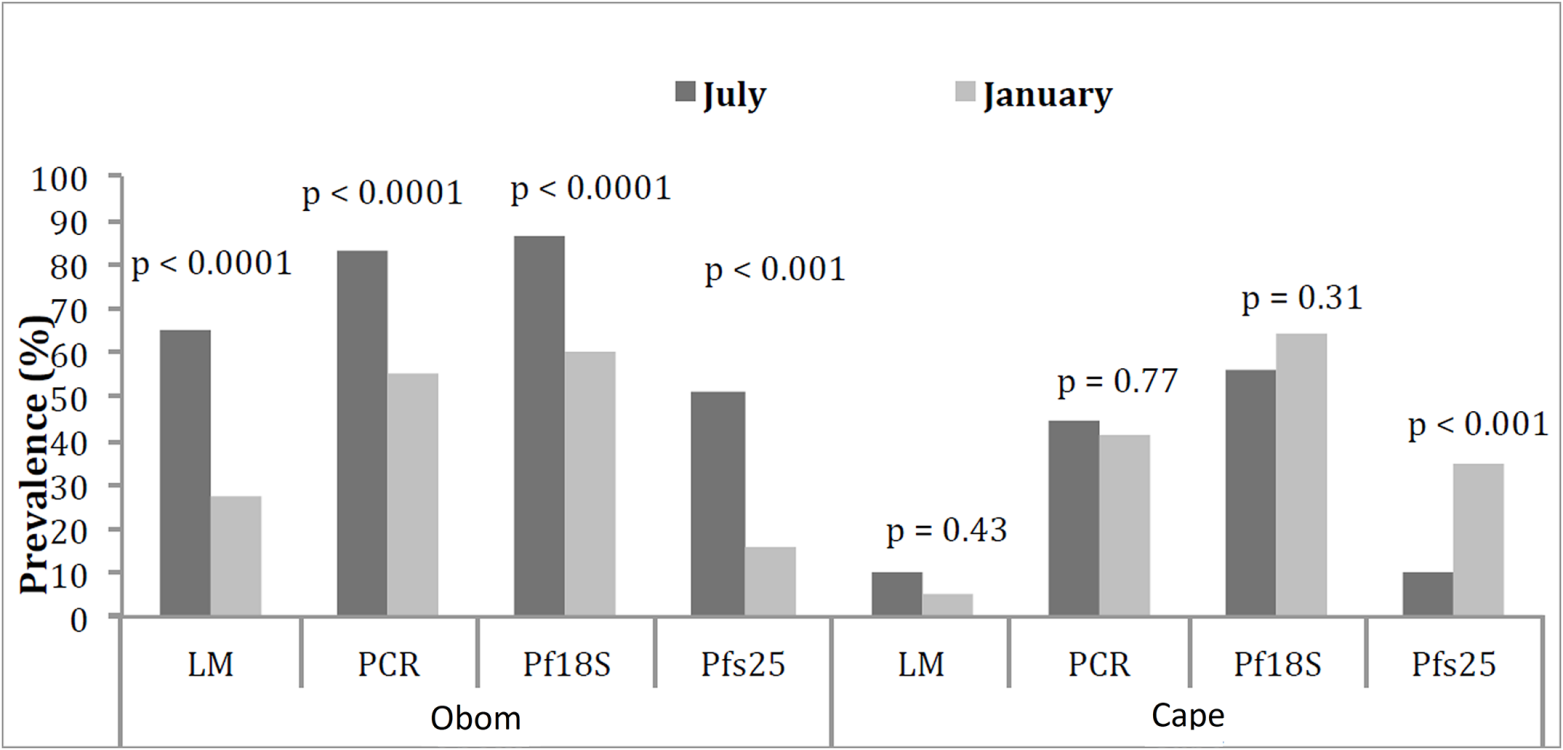
Asexual parasite and gametocyte prevalence in Obom and Cape Coast (Cape). Parasite prevalence in Obom and Cape Coast during the rainy and dry seasons as measured by microscopy (LM), conventional PCR (*Pf18S* rRNA, DNA amplification) and by qRT-PCR (*Pf18S* rRNA, transcript amplification) and submicroscopic parasite carriage determined using *Pfs25* qRT-PCR.

The prevalence of *P*. *falciparum* infected children identified by microscopic evaluation of thick blood smears was distinctly different in the two communities. In Obom parasite prevalence by microscopy was significantly higher in the rainy season (65%, 95%CI: 50–80)) than the dry season (28%, 95%CI: 13–42) (P <0.0001), while in Cape Coast, there was no significant difference between the seasons,10% (95%CI: 0–12) and 6% (95%CI: 0–13) in the rainy and dry seasons, respectively (Fig 1). As anticipated, both PCR and qRT-PCR analysis of *Pf18s rRNA* detected much higher parasite prevalence in both communities, when compared to prevalence by microscopy (Fig 1, Table C in S1 File), reaching a maximum of 86% in Obom and 56% in Cape Coast during the rainy season when measured by qRT-PCR. Surprisingly, the prevalence measured by qRT-PCR in Cape Coast during the dry season was higher than, but not significantly different (P = 0.31) when compared to the rainy season (Fig 1, Table A in S1 File) while in Obom the prevalence decreased significantly (p < 0.0001) from 86% in the rainy season to 60% in the dry season (Fig 1, Table A in S1 File). The reason for this difference is unknown, but could be due to the difference in malaria transmission intensity between the two sites. In Obom, the gametocyte prevalence of 51% in the rainy season reduced to 16% in the dry season, while in Cape Coast it increased from 10% in the rainy season to 35% in the dry season.

In Cape Coast, parasite prevalence estimated by PCR was 34% (rainy) and 35% (dry) higher than microscopy estimates and by qRT-PCR it was 46% (rainy) and 58% (dry) higher than microscopy (Fig 1, Table C in S1 File). This suggests that for every child that tested positive by microscopy, there were between four to eleven children that had low density infections that were missed. The submicroscopic parasite prevalence estimated by qRT-PCR was 12% (rainy season) and 23% (dry season) greater than PCR in Cape Coast (Table C in S1 File). Although parasite prevalence estimated by both PCR and qRT-PCR did not change appreciably between the rainy and dry season (P = 0.38, Two-sample test for equality of proportions) a pronounced increase by 3.5 fold was observed among submicroscopic gametocyte prevalence (P < 0.001, Two-sample test for equality of proportions) [Fig 1 and Table in S1A Table].

In the rainy season, microscopy underestimated parasite prevalence in Obom by 28% and 32% when compared to estimates determined by PCR and RT-PCR respectively. Although the difference did not seem large, it was significant (P < 0.05, Two-sample test for equality of proportions) [Table A in S1 File]. Similarly, in the dry season, microscopy underestimated parasite prevalence by 49% and 53% when compared to both PCR and qRT-PCR respectively. Parasite prevalence estimated by PCR and RT-PCR were similar at both time points and each reduced by 28% and 26% respectively in the dry season compared to the rainy season. The decrease in submicroscopic gametocyte prevalence in the dry season compared to the rainy season was double that recorded for total parasite prevalence (69%) [Fig 1 and Table A in S1 File].

Malaria transmission is dependent on the prevalence of mature gametocytes in an infection. During the rainy season, 59% of the 39 children in Cape Coast had submicroscopic infections (asexual parasite positive) that contained submicroscopic gametocytes, whilst 70% of the 37 children in Obom harbored submicroscopic both asexual parasites and gametocytes. The reverse trend was observed in the dry season where the prevalence of children harboring asexual parasites with gametocytes was higher in Cape Coast (72%) than in Obom (59%) [Table B in S1 File].

### Paired (children present at both time points) sample analysis

Paired samples from both Obom and Cape Coast revealed how many total children were asymptomatic parasite carriers at either time point. Real time RT-PCR analysis identified only 6% (2/32) of children in Obom and 14% (4/29) of children in Cape Coast to be free of an active infection in both seasons. As high as 44% (14/32) of the children in Obom and 31% (9/29) in Cape Coast were parasitaemic in all seasons (Table 2). Twenty-one percent (3/14) of children from Obom simultaneously harbored mature gametocytes in both the rainy and dry season. None of the children from Cape Coast harbored gametocytes during both the rainy and dry season (Table 2). Sixty seven percent of children who were parasitaemic in the rainy season but not the dry season harbored mature gametocytes in Obom; this was significantly higher than the 20% of the children in Cape Coast who were parasitaemic only in the rainy (but not the dry season) who harbored mature gametocytes (Table 2). More participants in Cape Coast were parasitaemic in the dry season than in Obom. Six out of 11 (55%) of these parasitaemic children harbored mature gametocytes in Cape Coast, whist only a child (25%) out of the 4 children who were parasitaemic in the dry season in Obom harbored mature gametocytes (Table 2).

**Table 2:** Parasite (Asexual and gametocyte) prevalence in paired samples.

Visit	*Pf*18S (Obom) (% of n)	*Pf*18S (Cape) (% of n)	P value	Pfs25 (Obom)* (% of 18S)	Pfs25 (Cape)* (% of 18S)	P value
July +/ Jan. +	44	31	0.08	21	0	< 0.0001
July +/ Jan. -	38	17	0.002	67	20	< 0.0001
July -/ Jan. +	13	38	< 0.0001	25	55	< 0.0001
July -/ Jan. -	6	14	0.099	0	0	

July +, positive / infected in July; July -, negative / not infected in July; Jan. +, positive / infected in January; Jan. -, negative / not infected in January; n, total count; *Pf18S*, total parasite. Pfs25*, total mature gametocyte (qRT-PCR on samples with and without microscopic gametocytes). Paired samples in for each sites (n = 32 for Obom, n = 29 for Cape Coast). *Pf*18S is reported as a % of the total number of paired children but *Pfs25* is reported as a % of *18S* positive (parasitaemic) children.

## Discussion

Microscopy, the gold standard for malaria diagnosis has the limitations of requiring expertise, electricity and has low detection sensitivity, ~50 parasites/μL blood by expert microscopists [28,29]. This low sensitivity can be a major problem in low transmission settings and in detecting asymptomatic infections. The introduction of malaria rapid diagnostic tests (RDTs) revolutionized malaria diagnosis, as they provide quick results without the need for specialized equipment or personnel. The RDTs are widely used in managing malaria in resource constraint settings and also used in mapping *Plasmodium* infections during community surveys [30]. Yet, the relatively low sensitivity (> 100 parasites/μL) of RDT [31], recent identification of parasites which have deletions in the *Plasmodium falciparum histidine rich protein 2* (*pfhrp2)* gene [32,33] and the persistence of the *P*. *falciparum* Histidine Rich Protein 2 (HRP2) antigen have hampered the usefulness of rapid PfHRP2 based malaria diagnostics. Parasite prevalence can also be determined using molecular tools including polymerase chain reaction (PCR), which has a limit of detection (LOD) of 1–5 parasites/μL of blood, reverse transcriptase PCR (RT-PCR) and real time RT-PCR (qRT-PCR), which both have limits of detection approaching > 0.5 parasites/μL of blood to detect the *Plasmodium* small subunit ribosomal *18S rRNA* gene [26,34,35] or transcript [36], respectively. These molecular tools can also be used to differentiate between the parasite species [28,37–40]. However, these methods are expensive and require technical expertise, electricity, cold storage and specialized reagents.

Gametocyte carriage is an essential component of malaria transmission in any community. However, public health programs do not prioritize monitoring gametocyte densities, due to the need for parasite RNA to differentiate gametocytes from the total parasite population. Historically, gametocytes have been detected using light microscopy, with sensitivities of about 20 mature gametocytes/μL of blood [41,42]. However, in most individuals, gametocyte carriage is usually at submicroscopic densities and requires sensitive molecular tools, to detect stage-specific transcripts that are not as abundant as the *18s rRNA*. The most common tools used to accurately estimate gametocyte carriage include reverse transcriptase PCR (RT-PCR), real time reverse transcriptase PCR (qRT-PCR) and quantitative nucleic acid amplification (QT-NASBA) [41,43–45]. The main transcripts used to monitor gametocyte densities and prevalence include the female mature gametocytes specific transcript *Pfs25* [46,47], and *Pfs230* paralogue, *Pfs230p*, that detects mature male gametocytes [48,49] and *Pfs16*, which is present in all gametocyte stages [50]. Similarly, *Pfg377* a female specific transcript is used to determine gametocyte diversity [51].

Gametocyte production has been suggested to differ in areas with varying microscopic parasite prevalence [43]. Thus, to monitor the infectious reservoir in two communities with different malaria transmission profiles we assessed both the gametocytes as well as the asexual parasitemia to determine the relationship. We also compared three different malaria parasite detection tools to identify the most effective.

### Detection tools

Microscopy, was confirmed to have significantly lower sensitivity at detecting *P*. *falciparum* parasites than PCR and qRT-PCR in both high and low parasite intensity settings (Table S1 Table 1C), as has previously been established [52–54]. Surprisingly, although RDT positivity rate is not a true indicator of parasite prevalence as PfHRP2 antigen persistence and the presence of parasites with deletions in the *Pfhrp2* gene increase the incidence of false positive or negative test results [55,56], in this study it provided a better estimate of parasite prevalence than microscopy in both sites. The RDT positivity rates were significantly higher than parasite prevalence estimated by microscopy but similar to parasite prevalence estimated by PCR in both sites, suggesting that the HRP2 antigen levels detected by the RDT were most likely due to antigens produced by active low level infections that were undetectable by microscopy rather than past exposure to parasites. This similarity between RDT and PCR results suggests that substituting microscopy for the much simpler RDT could provide a more accurate estimation of *P*. *falciparum* prevalence when screening asymptomatic individuals. In Cape Coast, parasite prevalence estimated by PCR increased by 27% in the wet season and 56% in the dry season when assayed by qRT-PCR, which is consistent with previous reports that real time RT-PCR has much higher sensitivities at detecting malaria parasites compared with PCR [41,42,44]. It is likely that this difference in *P*. *falciparum* estimation by PCR and qRT-PCR was only observed in Cape Coast, not Obom, because the parasitemias were lower, with some densities below the detection limit of PCR during both seasons. In Cape Coast the average parasitemia was 1015 parasites/μL in the wet season and only 530 parasites/ μL in the dry season compared to Obom where the average parasitemia was above 1750 parasites/ μL in both seasons. More studies with a larger population and in communities with varying malariometric indices are needed to define the cut-off parasitemia for the use of PCR or possibly RDT to assess asymptomatic cohorts.

### Seasonal differences in parasite prevalence

Another major difference between Cape Coast and Obom was the seasonal change in both total parasite and gametocyte prevalence. In Obom there was a significant reduction in parasite prevalence in the dry season by all the three assessment methods as well as a decrease in gametocyte prevalence by *Pfs25* qRT-PCR. In contrast, In Cape Coast although microscopic parasite prevalence decreased by 50%, there was no difference in submicroscopic parasite prevalence in Cape Coast (P > 0.05) between the two seasons. The difference observed by microscopy could be due to the 50% decrease in the average parasitemia in Cape Coast in the dry season, which decreased the number of individuals with parasitemias above the threshold for microscopy but still above the level needed for PCR or RT-PCR. Despite the decrease in total parasite prevalence the gametocyte prevalence significantly increased from 10% in the wet season to 35% in the dry season.

Differences in the dynamics of the mosquito populations in the wet and dry seasons in Obom and Cape Coast could contribute to differences in parasite prevalence. Since parasite prevalence as measured by RT-PCR is similar in both communities in the dry season it is possible that there are similar numbers of circulating mosquitos, but that during the wet season this population only increases significantly in Obom. Obom is a more rural setting with more areas for water to pool creating a seasonal increase in mosquito breeding sites. However, the decrease in Obom could also have been related to the National Malaria Control Program that distributed insecticide treated bed nets in the community four months prior to the January sampling, which was not simultaneously carried out in Cape Coast [57]. Additional longitudinal testing in successive years is needed to evaluate the observed seasonal parasite prevalence patterns.

### Gametocyte prevalence

The Control intervention in Obom could also have decreased the gametocyte prevalence in the community, but this does not explain the significant increase in gametocyte prevalence in Cape Coast in the dry season. Even in the wet season, prior to the control intervention the gametocyte prevalence differed in the two communities. During the wet season in Obom 59% of the parasitemic individuals had gametocytes, while in Cape Coast there was lower parasite prevalence and parasitemia and only 17% were gametocytemic. In the dry season the parasite prevalence remained the same in Cape Coast, while the average parasitemia decreased from 1000 parasites/μL to 500 parasites/μL, but the gametocyte prevalence increased so that 52% of the parasite carriers were gametocytemic. The reason for this seasonal increase is unclear and was not observed in Obom, which in the dry season had a similar parasite prevalence to Cape Coast, although in Obom the average parasitemia was much higher, 2300 parasites/μL. It is possible that parasite strains with higher conversion rates are selected for during this time or that conditions in the host during these periods influenced sexual differentiation and maturation allowing continuous transmission as suggested by Okell and colleagues [43]. However, the ratio of gametocyte carriers to parasitemic individuals was even higher in Obom during the high transmission wet season (59%) than it was in Cape Coast during the dry season and is more in line with suggestions that high parasitemia enhances gametocyte production [58]. Reconciling this data requires additional longitudinal studies to confirm the patterns as well as experiments to directly evaluate of the number of gametocyte-committed rings and their in vivo progression to circulating stage V gametocytes 10–12 days later.

### Prevalence in paired samples

Using paired samples allowed the direct evaluation of the number of individuals that were parasitemic at one or more collection times. These results indicate that 87% (95% CI: 78–96) of the individuals at both sites were parasitemic at least once within the 6 month study period and that a high number of the participants at both locations harboured an asymptomatic infection at any one time during both the wet and dry season, with the peak season in Cape Coast registering the lowest prevalence of 48% (95% CI: 29–68). Such high prevalence asymptomatic infections have previously been reported during the dry season in Northern Ghana, where more than 50% of the participants (children under 12 years) harboured submicroscopic infections [59,60] and suggests the need for considering the expansion of SMC control programs throughout Ghana. Again, the pattern of gametocyte carriage differs between sites. In Cape Coast none of the individuals that were parasite positive at both visits were gametocyte carriers, while in Obom 20% of the individuals that were parasitemic at both collections had gametocytes. Consistent with the higher gametocyte prevalence observed in the wet season in Obom and the dry season in Cape Coast more that 55% of the individual that only had parasites during these collections were gametocytemic. It has been suggested that 20–50% of the overall malaria transmission result from submicroscopic infections [43] therefore these high levels of gametocyte prevalence are likely to be making a substantial contribution to transmission and additional work is needed to understand underlying factors involved in the complex seasonal pattern observed in this study.

## Limitation

The absence of active or passive follow up of the study participants prevented the observation of any possible progression of an asymptomatic *P*. *falciparum* infection into a symptomatic infection. Only a small number of children were sampled in this study. Additional longitudinal studies with a larger sample set over sequential years are needed to confirm the observed distinct seasonal patterns of asexual parasite and gametocyte prevalence.

## Conclusion

Parasite carriage in southern Ghana is much higher than previously classified, with majority of the infections presenting as asymptomatic submicroscopic infections. Parasite prevalence estimated by PCR and qRT-PCR is significantly different only in low parasite density settings, thus for an accurate map of parasite carriage in Ghana, PCR or possibly RDT can be used in high parasite density settings and qRT-PCR used in low parasite density settings. Communities with varying transmission patterns also exhibited marked differences in the seasonal pattern of mature gametocyte carriage and the factors contributing to this need to be evaluated further.

## Supporting information

S1 File Table A: Comparison of parasite prevalence between the two seasons in both sites, Table B: Comparison of parasite prevalence between the two sites at each season and Table C: Sensitivities of detection tests at assessing parasite prevalence.
(DOCX)
