# Supplementary material for: Seasonal variations in Plasmodium falciparum parasite prevalence assessed by varying diagnostic tests in asymptomatic children in southern Ghana
Source: PLoS One. 2018 Jun 15;13(6):e0199172. doi: 10.1371/journal.pone.0199172 (PMC6003688; doi:10.1371/journal.pone.0199172)
Supplement: S1 File — Table A: Comparison of parasite prevalence between the two seasons in both sites, Table B: Comparison of parasite prevalence between the two sites at each season and Table C: Sensitivities of detection tests at assessing parasite prevalence. (DOCX) [file pone.0199172.s001.docx]

# S1 file

**Table A**: Comparison of parasite prevalence between the two seasons in both sites.

| Site & visit | Microscopy | | | PCR | | | *Pf18S rRNA* | | | *Pf25* | | |
| --- | --- | --- | --- | --- | --- | --- | --- | --- | --- | --- | --- | --- |
|  | % | X^2^ | P value | % | X^2^ | P value | % | X^2^ | P value | % | X^2^ | P value |
| Cape Coast July | 10 | 0.61 | 0.43 | 44 | 0.08 | 0.77 | 56 | 1.02 | 0.31 | 10 | 17.6 | <0.001 |
| Cape Coast January | 6 |  |  | 41 |  |  | 64 |  |  | 35 |  |  |
| Obom July | 65 | 26.1 | <0.0001 | 83 | 17.04 | <0.0001 | 86 | 15.6 | <0.0001 | 51 | 30.5 | <0.001 |
| Obom January | 28 |  |  | 55 |  |  | 60 |  |  | 16 |  |  |

Parasite prevalence in Obom and Cape Coast during the rainy and dry seasons as measured by microscopy (LM), conventional PCR (*Pf18S* *rRNA*, DNA amplification), qRT-PCR (*Pf18S* *rRNA*, transcript amplification) and submicroscopic parasite carriage determined using *Pfs25* qRT-PCR.

**Table B**: Comparison of parasite prevalence between the two sites at each season

| Site & visit | Microscopy | | | PCR | | | *Pf18S rRNA* | | | *Pf25* | | |
| --- | --- | --- | --- | --- | --- | --- | --- | --- | --- | --- | --- | --- |
|  | % | X^2^ | P value | % | X^2^ | P value | % | X^2^ | P value | % | X^2^ | P value |
| Cape Coast July | 10 | 62.2 | <0.0001 | 44 | 31.2 | <0.0001 | 56 | 20.4 | <0.0001 | 10 | 40.9 | <0.001 |
| Obom July | 65 |  |  | 83 |  |  | 86 |  |  | 51 |  |  |
| Cape Coast January  Obom January | 6 | 15.6 | <0.0001 | 41 | 3.4 | 0.066 | 64 | 0.19 | 0.66 | 35 | 10.5 | 0.001 |
|  | 28 |  |  | 55 |  |  | 60 |  |  | 16 |  |  |

Parasite prevalence in Obom and Cape Coast during the rainy and dry seasons as measured by microscopy (LM), conventional PCR (*Pf18S* *rRNA*, DNA amplification), qRT-PCR (*Pf18S* *rRNA*, transcript amplification) and submicroscopic parasite carriage determined using *Pfs25* qRT-PCR.

**Table C**: Sensitivities of detection tests at assessing parasite prevalence

|  | Microscopy | | PCR | | *Pf18S rRNA* | |
| --- | --- | --- | --- | --- | --- | --- |
| Site & visit | Prevalence % | Sensitivity % | Prevalence % | Sensitivity % | Prevalence % | Sensitivity % |
| Cape Coast July | 10 | 18 | 44 | 79 | 56 | 100 |
| Cape Coast January | 6 | 9 | 41 | 64 | 64 | 100 |
| Obom July | 65 | 76 | 83 | 97 | 86 | 100 |
| Obom January | 28 | 47 | 55 | 92 | 60 | 100 |

Parasite prevalence obtained by qRT-PCR of Pfs18S rRNA at each time point was set at 100% and used as the reference calibrator for parasite prevalence estimated by microscopy and PCR.
